# No evidence for a bioenergetic advantage from forced swimming in rainbow trout under a restrictive feeding regime

**DOI:** 10.3389/fphys.2015.00031

**Published:** 2015-02-06

**Authors:** Peter V. Skov, Ivar Lund, Alexandre M. Pargana

**Affiliations:** Section for Aquaculture, The North Sea Research Centre, DTU Aqua, Technical University of DenmarkHirtshals, Denmark

**Keywords:** water current, feed conversion, oxygen consumption, nitrogen excretion, swimming, metabolic rate, fuel use

## Abstract

Sustained swimming at moderate speeds is considered beneficial in terms of the productive performance of salmonids, but the causative mechanisms have yet to be unequivocally established. In the present study, the effects of moderate exercise on the bioenergetics of rainbow trout were assessed during a 15 week growth experiment, in which fish were reared at three different current speeds: 1 BL s^−1^, 0.5 BL s^−1^ and still water (≈ 0 BL s^−1^). Randomly selected groups of 100 fish were distributed among twelve 600 L tanks and maintained on a restricted diet regime. Specific growth rate (SGR) and feed conversion ratio (FCR) were calculated from weight and length measurements every 3 weeks. Routine metabolic rate (RMR) was measured every hour as rate of oxygen consumption in the tanks, and was positively correlated with swimming speed. Total ammonia nitrogen (TAN) excretion rates showed a tendency to decrease with increasing swimming speeds, yet neither they nor the resulting nitrogen quotients (NQ) indicated that swimming significantly reduced the fraction of dietary protein used to fuel metabolism. Energetic budgets revealed a positive correlation between energy expenditure and the current speed at which fish were reared, fish that were forced to swim and were fed restrictively consequentially had poorer growth and feed utilization. The results show that for rainbow trout, water current can negatively affect growth despite promoting minor positive changes in substrate utilization. We hypothesize that this may be the result of either a limited dietary energy supply from diet restriction being insufficient for both covering the extra costs of swimming and supporting enhanced growth.

## Introduction

The use of a moderate water current, to elicit a low level of sustained aerobic exercise, can have beneficial effects on several parameters of productivity and welfare of farmed salmonids (McKenzie et al., 2012; Davison and Herbert, 2013). Swimming induced benefits can be divided between physical effects of water current and derived behavioral changes as well as direct physiological benefits from swimming. Salmonids tend to exhibit schooling behavior when subjected to a water current, resulting in less agonistic interactions, reduced hierarchy formation, and lower stress levels (Christiansen and Jobling, 1990; Adams et al., 1995; Brännäs, 2009). Regardless of schooling, dominant fish may display less aggression to compensate for the higher energetic costs of swimming, which in turn increases the opportunities for subordinate individuals to feed (Christiansen and Jobling, 1990; Davison, 1997), and promotes growth by lowering stress levels in all categories of fish (Adams et al., 1995; Davison, 1997). While fish in schools obtain hydrodynamic benefits and swim steadily (East and Magnan, 1987), fish kept in still water display a much higher degree of spontaneous or erratic activity (Brännäs, 2009), with frequent changes in speed, acceleration, turning rate, and direction, a pattern that can be 2.5 to 6-fold more costly in energetic terms (Krohn and Boisclair, 1994; Steinhausen et al., 2010).

Cost of transport is often used as an argument for the benefits of swimming, regardless of the need for transportation. From a purely bioenergetic perspective, there can be no doubt that swimming is more costly than non-swimming. Still, evidence for enhanced growth performance (East and Magnan, 1987; Houlihan and Laurent, 1987; Jobling et al., 1993; Jørgensen and Jobling, 1993; Davison, 1997) suggests that the additional costs of swimming are more than met by compensatory gains. The mechanisms underlying such effects seem to result from a number of physiological changes induced by the presence of water current. Exercise can influence body composition through changes in protein and lipid deposition, although results from various studies are sometimes contradictory (Nahhas et al., 1982; East and Magnan, 1987; Houlihan and Laurent, 1987; Christiansen et al., 1989; Davison, 1997). Swimming may cause a reduction in the cost of living through changes in standard metabolic rate (Skov et al., 2011) while increased levels of circulating growth hormone and insulin-like growth factor (Sumpter, 1992; Davison, 1997; Deschamps et al., 2009) may influence protein turnover. It is known that fish peripheral tissues have a poor ability to clear a glucose load from circulation, which has led some authors to refer to teleost fish as being glucose intolerant (Moon, 2001). However, exercise has been shown to have stimulatory effects on glucose utilization in rainbow trout red muscle (West et al., 1993; Felip et al., 2012). This is achieved in part by an increased glucose uptake by muscle cells (Felip et al., 2012) mediated by increased AMPK (Magnoni et al., 2012, 2014). Although the overall contribution from glucose in oxidative processes remains low, it could represent an avenue of improved energetic efficiency, since the extra costs of swimming could be counterbalanced by the efficient utilization of a fuel source that would be otherwise physiologically unavailable.

The purpose of the present study was to investigate the effects of water current on the growth performance and bioenergetics of rainbow trout maintained on a restricted diet, as a way to better expose the effects of exercise and prevent that differences in energy requirements be compensated and masked by an increased or differences in feed intake. Three different regimes were tested: a still water control group, a 0.5 body length per second intermediate current speed group, and a 1 body length per second group. The selected temperature was 15°C, considered to be the optimum temperature for growth in rainbow trout (Sumpter, 1992). By use of a rearing tank system that allowed instantaneous measurements of oxygen uptake, any differences in growth rate or feed conversion ratio (FCR) could be interpreted in the light of the relative energetic efficiency of the fish at different conditions. Energetic budgets were calculated based on values of feed intake and routine metabolic rates. In order to asses any correlation between exercise regime and protein retention, rates of ammonia excretion were measured and related to oxygen consumption in order to detect any differences in the rate of amino acid deamination.

## Materials and methods

### Animal husbandry

Fish were obtained from a commercial fish farm (Funderholme Dambrug) and quarantined for 2 weeks in 15ppt sea water. Following quarantine, fish were randomly distributed in groups of 100 individuals among twelve circular polyethylene tanks with a water volume around 600 L, identical to those previously described by Larsen et al. (2012). Each tank had an internal cylindrical PVC column, creating a circular canal within the tank in which water could circulate. The tanks were connected to a common water supply within a recirculation bio-filtered system that delivered a 50 L min^−1^ flow of aerated freshwater at a constant temperature of 15°C to each tank by means of a centrifugal pump (Grundfos TP 25-90/2, Grundfos DK A/S, Bjerringbro, Denmark) fitted to each tank. Water quality parameters (NO^−^_3_, NO^−^_2_, NH_3_/NH^+^_4_, pH) were monitored daily and did not exceed safe levels throughout the course of the study (NO^−^_3_ < 100 mg l^−1^, NO^−^_2_ 0–1 mg l^−1^, total ammonia NH_3_/NH^+^_4_ 0–1 mg l^−1^). The inlet consisted of a vertical PVC pipe with a row of apertures fixed to the inner wall of the tank. The speed of the current could be controlled through the selection of aperture size and number and the direction at which the apertures of the inlet pipe were oriented relatively to the swimming canal. Water flow was adjusted in each tank so that three groups of four tanks were set with different current velocities while receiving the same volume of water over time. The different rearing conditions used were: no current (O); low current (LC) at 0.5 body lengths per second (BL s^−1^); and high current (HC) at 1 BL s^−1^. The velocity of the water current was measured twice per week using a propeller flow-meter (OTT Hydrometrie Z30, Germany) in the center of the swimming canal at three different depths. Minor adjustments were made by changes in water flow to the tank, while larger adjustments over time (as fish grew) were achieved by changing to inlet pipes with smaller apertures. In tanks with no water current the vertical inlet pipe was oriented so that the formation of a circular flow pattern never exceeded 0.1 BL s^−1^, while water exchange were maintained at similar rates as in the other tanks.

Photoperiod was maintained at 14 h: 10 h light: dark (lights on at 7:00) throughout the experiment. Feeding was delivered by automatic belt feeders for periods lasting 8 h (between 8:00 and 16:00). The feeding regime was set up from Rasmussen and From's (1991) growth model based upon energy flow and partitioning parameters estimated from tank experiments with rainbow trout. This model defines feeding level *f* as the fraction eaten of the maximum quantity which could be eaten (0 ≤ *f* ≤ 1). The feeding level chosen for the growth trial was 0.84, corresponding to an initial daily ration of 1.3% of the estimated tank biomass and gradually reduced to a more restricted regime of 0.9% of the biomass. The feed consisted of 3 mm and later 4.5 mm extruded pellets (42–47% protein, 28–32% fat, 12–13% carbohydrate; EFICO Enviro 920, Biomar A/S, Brande, Denmark). The transition from 3 mm to 4.5 mm pellets took place between days 27 and 31 in a progressive way, with increments of 20% day^−1^ of the bigger sized pellets. Feces and debris were removed through a central drain in the bottom of the tank connected to a swirl separator, and any uneaten pellets were deducted from the daily ration. All use of animals for these experiments was in accordance with Danish and EU legislation.

### Growth performance

Overall growth performance was calculated from a 15 week period (95 growth days), where fish mass and length were measured at five 21-day intervals, each consisting of 19 feeding days followed by 2 full days of fasting (the last interval had two extra days). Measurements were performed on day 21 and feeding was resumed the following day. Prior to manipulation, the fish were anesthetized with 2-phenoxyethanol (0.5 ml l^−1^). The total biomass of each tank was measured, and the fish were counted to derive the mean mass for the fish in each tank. Photographs were used to measure the fork-length, with the aid of the software IrfanView 4.32. By the end of each measuring procedure the fish were returned to their tanks and current speed was adjusted according to the new mean body length for each tank. The total biomass in each tank was used to calculate the daily amount of feed for the following 19-day feeding period.

The specific growth rate (SGR) for each feeding period was calculated per tank on the total biomass, as:

(1) $$SGR=100\times \left(\frac{\ln final\;biomass\;-\ln initial\;biomass}{number\;of\;days}\right)$$

Only feeding days were included in the calculation of SGR and the weights of fish that died during the period were included.

The FCR was calculated per tank as:

(2) $$FCR=\frac{feed\;intake}{biomass\;increase}$$

The mean of the SGR and FCR values from all five intervals was used as the overall SGR and FCR.

### Energetic budgets

Energetic budgets were calculated as previously described by Larsen et al. (2012) and McKenzie et al. (2012) for periods of similar growth for all treatment groups. Mean fish mass was calculated from the total biomass and number of individuals in each tank at each weighing-day and plotted against time to fit exponential growth curves for the entire growth trial. The growth curves were then used to identify periods when the fish had similar mean mass in all tanks. The selected time frame corresponds to the period when the fish grew from 250 to 350 g. For each of these days, feed intake and oxygen consumption were used to calculate several energetic parameters (see **Figure 2**).

### Standard metabolic rate

In order to assess the amount of energy required for maintenance by the fish from each current speed, the resting oxygen consumption was determined using computerized intermittent flow through respirometry (Steffensen, 1989; Skov et al., 2011). During a period of 2 weeks immediately after the conclusion of the growth trial, four individuals were successively sampled from each tank, with 24 h between one tank and the next (mean body mass for each group, grams ± SE: HC 411 ± 19; LC 400 ± 17; OC 402 ± 16; N=48 (16 per water current). Prior to experimentation, feeding was suspended for 48 h in the tank to be sampled, and resumed once fish had been removed. The fish were netted from the tank, weighed and transferred to 8.66 L Plexiglas respirometers immersed in a 60 L bath at 15°C, and covered with a sheet of black opaque plastic to prevent any external visual disturbance. Aerated and UV treated (9 W UV-C, AquaCristal GmbH, Neuhofen, Germany) water was recirculated between the experimental setup and a 600 L reservoir (40% replacement by volume daily) via a trickling filter. Oxygen concentration in the water was measured every second using fiber optic oxygen sensors (Fibox 3, Precision Sensing GmbH, Regensburg, Germany) collected by automated respirometry software (AutoResp, Loligo Systems, Tjele, Denmark). Fish were allowed to acclimate to the novel conditions overnight, and then oxygen consumption measurements were performed in 15 min cycles comprising an 8 min flushing period, a 1 min waiting period and a 6 min measurement period.

The software used the slope of oxygen decline during the measurement period to calculate the oxygen consumption as mg O_2_ kg^−1^ h^−1^. These data were sorted in 10 mg intervals to create a frequency distribution, and standard metabolic rate (SMR) was calculated as described previously (Skov et al., 2011).

### Routine metabolic rate

The routine metabolic rate (RMR) of the fish was measured every hour as rate of oxygen uptake in each tank, using automated stop-flow respirometry (McKenzie et al., 2007, 2012). Tanks in the system alternated between periods of flow-through and recirculation without input of aerated filtrated water. Once per hour on the hour, a 3 way actuater (ER20, Valpes, Moirans, France) switched water flow to recirculation for a period of 10 min. During this time, the decline in water oxygen concentration was measured by oxygen electrodes (Oxyguard Standard, Oxyguard International A/S, Birkerød, Denmark) and recorded on a PLC data logger every 20 s during the periods where there was no renewal of water in the tanks. An emergency oxygen release system was present in each tank, to ensure that water *S*O_2_ never fell below 70% at any time.

The data was sorted per tank in daily groups of 24 h and linear regression was performed on the decline in oxygen concentration. The gradient of oxygen decline, the total biomass of fish, and the total volume of water were then used to calculate oxygen consumption (*M*O_2_; mmol kg^−1^ h^−1^) by the fish in each tank.

The total biomass of the fish in each tank on each day was estimated from the SGR calculated for each 21-day period and the biomass measured at the start of the period, using:

(3) $$\ln biomass_{dayX}=\frac{SGR}{100}+\ln biomass_{day(X-1)}$$

where *biomass_dayX_* = biomass on day of interest.

For comparing *M*O over the selected range of body masses (250–350 g), the data was made mass-independent by standardizing to a body mass of 300 g, using the equation:

(4) $$M{\text{O}}_{2}{}_{\left(300\right)}=M{\text{O}}_{2}{}_{\left(m\right)}\times {\left(\frac{m}{300}\right)}^{1-A}$$

where *M*O_2(*m*)_ = oxygen consumption for a fish with a body mass *m*; *A* = allometric exponent describing the relationship between metabolic rate and body mass. A value of 0.8 was used, as in previous studies (Skov et al., 2011; McKenzie et al., 2012). This exponent is generally accepted as reasonably accurate estimator of the change in metabolic rate with size of several fish species, including rainbow trout (Bureau et al., 2002).

The hourly measures of *M*O_2_ during this period were summed to obtain the metabolic rate for the entire day and the average daily *M*O_2_ among tanks with the same current speed used for comparison between the different treatments. Furthermore, circadian rhythms in oxygen consumption were revealed over the 24 h cycle and minimum and maximum *M*O_2_ were found for each group.

Due to a failure of an O_2_ probe, the oxygen consumption measurements from one tank in the O group were discarded.

### Ammonia excretion and protein use

Water samples were collected on days 78, 80, and 82, for measurement of total ammonia nitrogen (TAN) excretion. Ammonia-N excretion (*M*NH_3_-N; mmol kg^−1^ h^−1^) was then related to the consumption of oxygen during the same period to calculate the extent of protein use, given by the nitrogen quotient (NQ).

The sampling procedure was automated and occurred simultaneously in the 12 rearing tanks, starting at 0:00 of each sampling day, and was repeated every 4 h corresponding to a total of 6 samplings per day. Twelve water pumps (Compact 300, Eheim, Germany) drew water from the tanks at the start and at the end of the 10 min periods of closed recirculation (6 s sampling, 0.5 l volume). Immediately after, 15 mL subsamples were centrifuged for 10 min at 3000 rpm, 0°C, and frozen at −20°C for later analysis. The increase in TAN content from initial to final samples corresponded to the excretion of ammonia in the tank.

Ammonia concentrations on the water samples were determined in duplicates using a spectrophotometric method based on the Danish standards for water analysis (DS, 1975).

The NQ was determined for each tank at each time as:

(5) $$NQ=\frac{M\text{N}}{M{\text{O}}_{2}}$$

where *M*N is total nitrogen excretion and was estimated directly from *M*NNH_3_-N, based on the work by Kajimura et al. (2004), who found that ammonia-N corresponds to 53–68% of the total nitrogen waste but, since the contribution of non-oxidized N-compounds was found to be on average 14%, this means that ammonia-N actually corresponds to 62–79% of the total pool of oxidized N-products. As the ammonia-N contribution is typically higher at low rations (Kajimura et al., 2004), the present study will use a NH_3_-N contribution of 79%. Hence, the new *M*N_(oxidized)_ was calculated from the measured *M*NH_3_-N:

(6) $$M{\text{N}}_{\left(\text{oxidized}\right)}=\frac{M{\text{NH}}_{3}-\text{N}}{79}\times 100$$

The result was divided by *M*O_2_ measured in the same period to obtain NQ.

The percentage use of protein to fuel metabolism was calculated by the ratio of NQ and 0.27, which represents the condition in which aerobic respiration is fuelled entirely by protein (Van Den Thillart and Kesbeke, 1978), and comparisons were made between the different treatments.

### Data analysis and statistics

Statistics were performed with SigmaPlot 11.0. The data were examined using One-Way analysis of variance (ANOVA). Holm-Sidak *post-hoc* tests were used to identify where any significant differences in an ANOVA had occurred. In all cases, *P* < 0.05 was the chosen level for statistical significance. All data are presented as mean values ± SE.

## Results

### Growth performance

**Figure 1:**
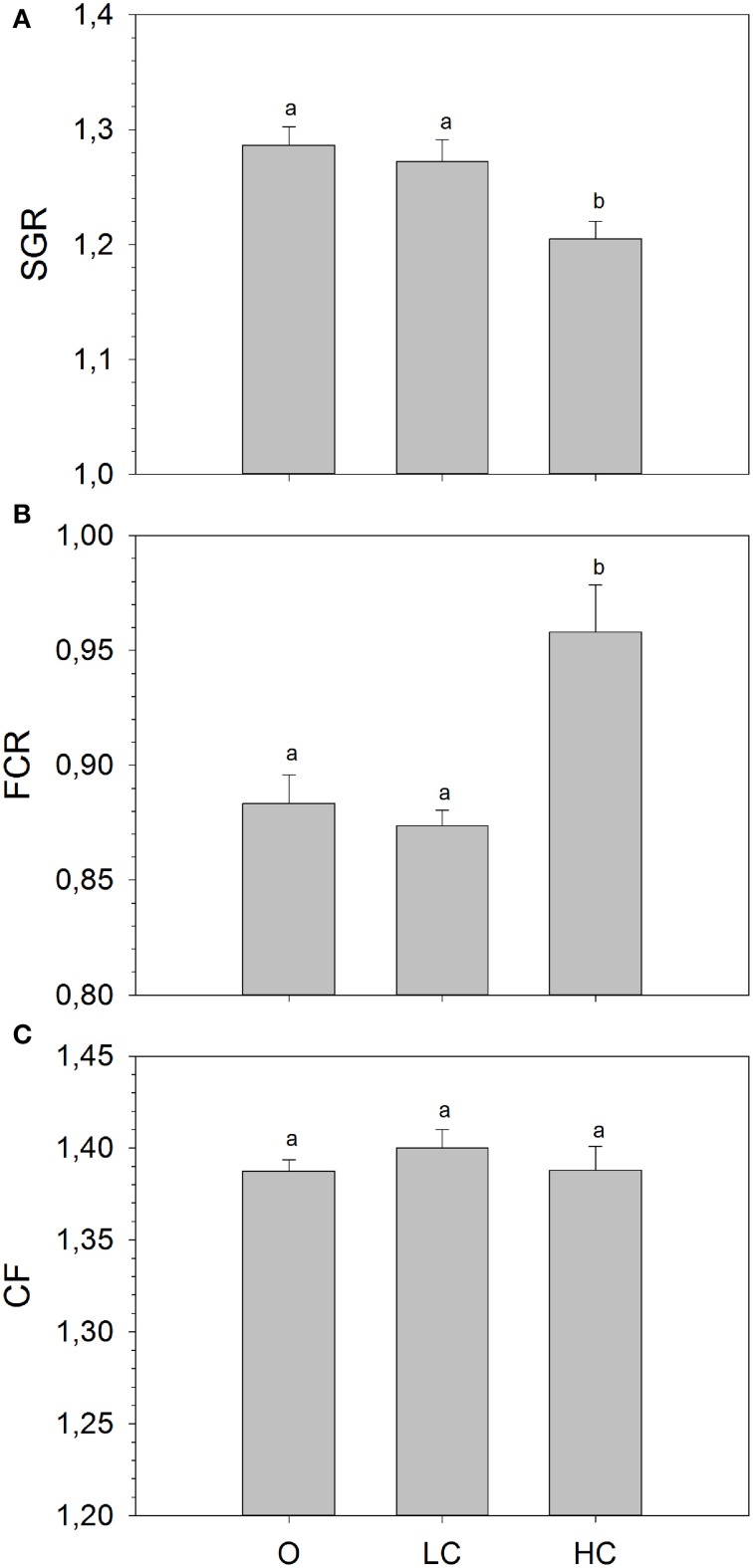
**(A)** Specific growth rate (SGR), **(B)** Feed conversion ratio (FCR), and **(C)** Condition factor (CF) calculated for the entire duration of the growth trial. High current, HC = 1 BL s^−1^; low current, LC = 0.5 BL s^−1^; and O = no current (mean ± SE, *n* = 4). Different superscripts denote significant differences between treatments.

The mean mass of fish (± SE) at the beginning of the growth study was: 110.7 ± 1.3 (O), 110.5 ± 1.7 (LC) and 112.7 ± 1.1 (HC) grams, with no statistical differences between the three experimental groups. At the end of the growth period, the mean mass of fish was: 376.8 ± 4.3 (O), 373.5 ± 4.2 (LC) and 349.8 ± 8.6 (HC). There were significant differences between mean SGR calculated for the entire growth period (Figure 1A), overall being significantly lower in HC than in LC and in O. There were no differences in mean SGR between LC and O. The mean FCR was significantly higher for the HC group compared to the LC and O. (Figure 1B). There were no differences in mean FCR between LC and O. No differences in condition factor were observed between treatments (Figure 1C).

**Figure 2:**
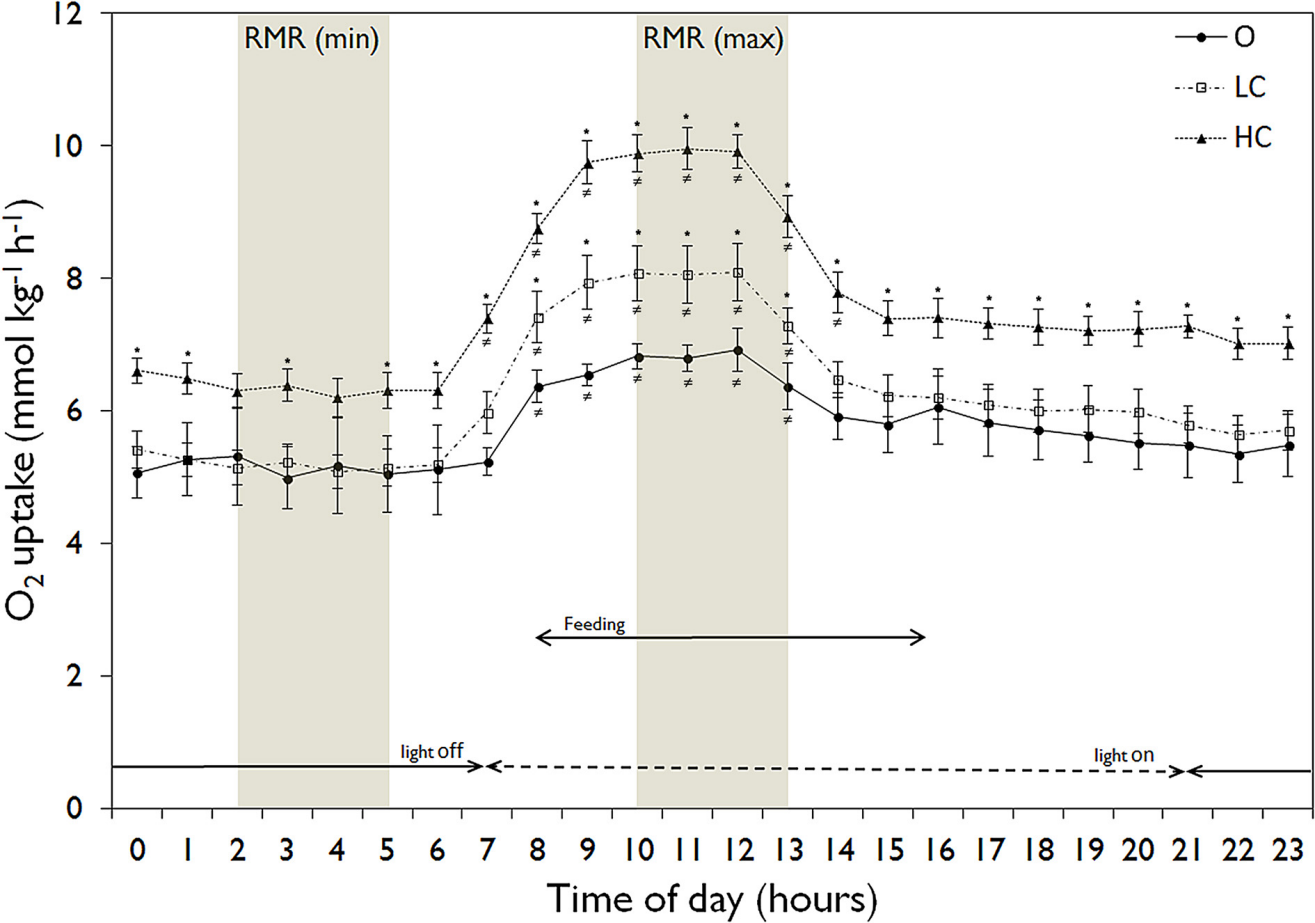
**Daily patterns of mean (± SE) hourly routine metabolic rate (RMR, normalized to 300 g) in the three experimental groups**. High current, HC = 1 BL s^−1^; low current, LC = 0.5 BL s^−1^; and O = no current (*n* = 4 in HC and LC; *n* = 3 in O). Light conditions and feeding hours indicated by arrows. The shaded areas show the time points corresponding to the minimum and maximum values for RMR. An asterisk denotes significant difference from corresponding value for the O group, while a not-equal to sign denotes a significant difference from minimum RMR values.

**Figure 3:**
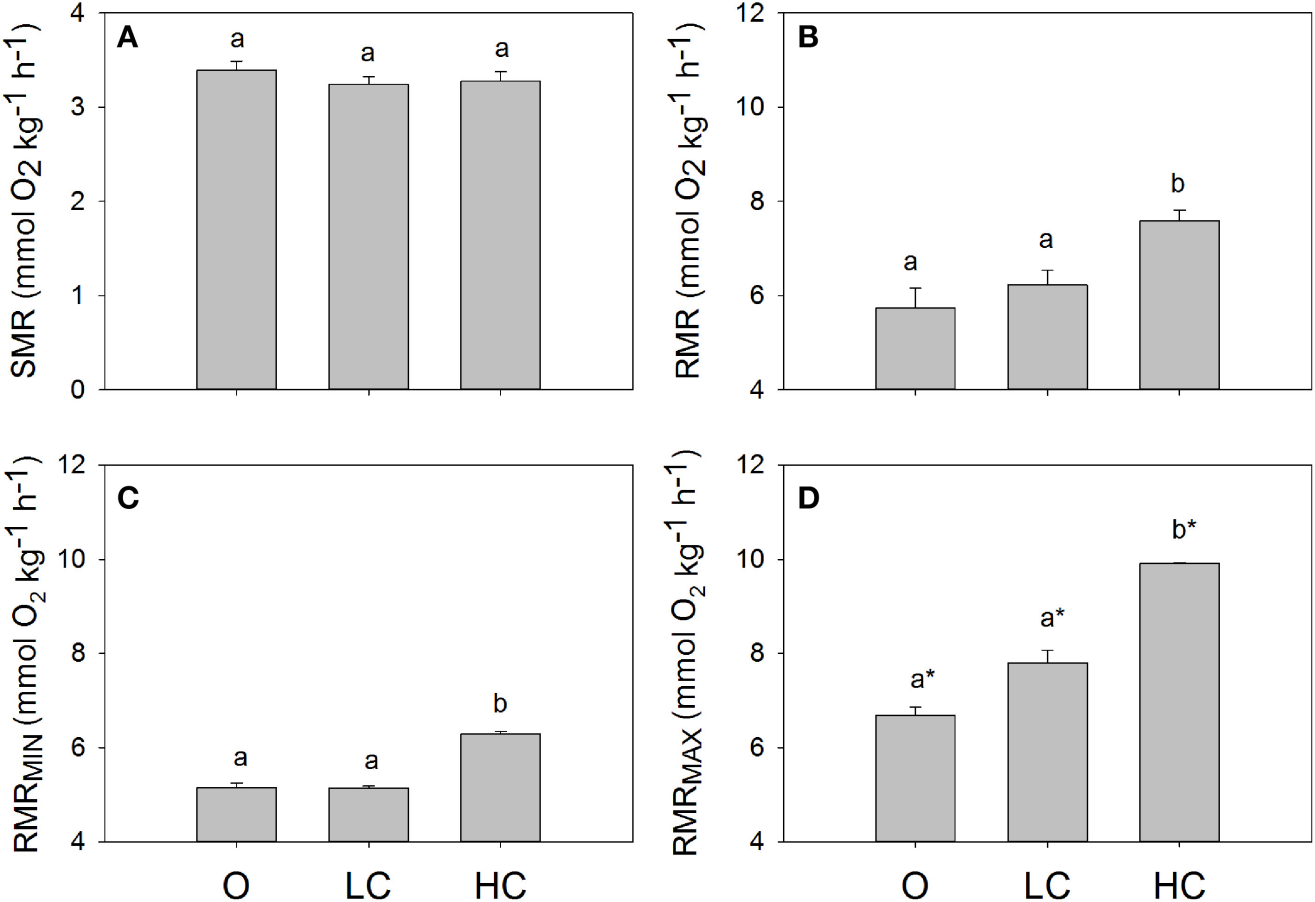
**Standard and routine metabolic rates for fish reared at different current speeds**. **(A)** Standard metabolic rate, SMR, **(B)** daily mean, **(C)** minimum and **(D)** maximum routine metabolic rates, RMR. Superscripts indicate significant differences between treatments. High current, HC = 1 BL s^−1^; low current, LC = 0.5 BL s^−1^; and O = no current (*n* = 4 in HC and LC; *n* = 3 in O). Superscript letters indicate significant differences between treatments, asterisks in **(D)** indicate significant differences from minimum RMR values.

All water current regimes displayed clear circadian cycles with large increases in MO_2_ during feeding hours and lower consumption levels during night time (Figure 2). There were no significant differences in SMR between current groups (Figure 3A). Daily average RMR was significantly higher in the HC group, compared to the LC and O group (Figures 2, 3B). The minimum (RMR_MIN_, Figure 3C) and maximum (RMR _MAX_, Figure 3D) RMR values occurred between 03:00 (O) and 04:00 (LC and HC) and between 11:00 (HC) and 12:00 (LC and O) respectively. For both minimum and maximum RMR values, the HC group was significantly higher than the O and LC groups.

### Energetic budgets

The energetic budgets for each experimental group during an increase in mean individual body mass from approximately 250–350 g is shown in Table 1.

**Table 1:** **Oxygen consumption, ammonia excretion rates, and nitrogen quotients (NQ) for groups of fish subjected to different water current velocities**.

	**O**	**LC**	**HC**
Oxygen consumption (mmol O_2_ kg^−1^ day^−1^)	140.7 ± 12.3^a^	158.0 ± 6.95^a^	192.5 ± 7.14^b^
Total ammonia nitrogen excretion (mmol Nkg^−1^ day^−1^)	20.8 ± 2.2	19.7 ± 2.3	22.7 ± 2.9
NQ	0.164 ± 0.006	0.126 ± 0.011	0.130 ± 0.019
%Protein use	60.8 ± 2.3	46.5 ± 4.1	48.1 ± 6.8

*Values are mean (±SE) from 3 days of measurements (days 78, 80, and 82 of the growth trial). High current, HC = 1 BL s^−1^; low current, LC = 0.5 BL s^−1^; and O = no current (n = 4 in HC and LC; n = 3 in O). Superscripts indicate significant differences between treatments*.

As the mean daily feed intake (g kg^−1^ day^−1^ ± SE) in this period was the same in all groups: 10.1 ± 0.04 (O), 10.0 ± 0.02 (LC), and 10.1 ± 0.04 (HC); this translated into a greater number of feeding days for the HC group to achieve a 100 g body mass increase. Feed loss was negligible in all tanks, a consequence of the restricted diet regime. The SGR calculated for this interval was significantly lower in HC than in LC and in O. Oxygen consumption in the selected period, and therefore total energy dissipation for metabolism, was significantly higher in HC than in LC and in O. Regarding the proportion of energy used relatively to the energy ingested, HC fish spent a significantly higher amount of energy than LC and O. The higher rate of energy utilization in the HC group meant that a significantly smaller proportion of the energy intake was apparently retained for allocation toward somatic growth. The gross cost of growth, i.e., the amount of energy required per gram of mass gained, was therefore higher in HC group compared to the other groups.

### Ammonia excretion and protein use

**Figure 4:**
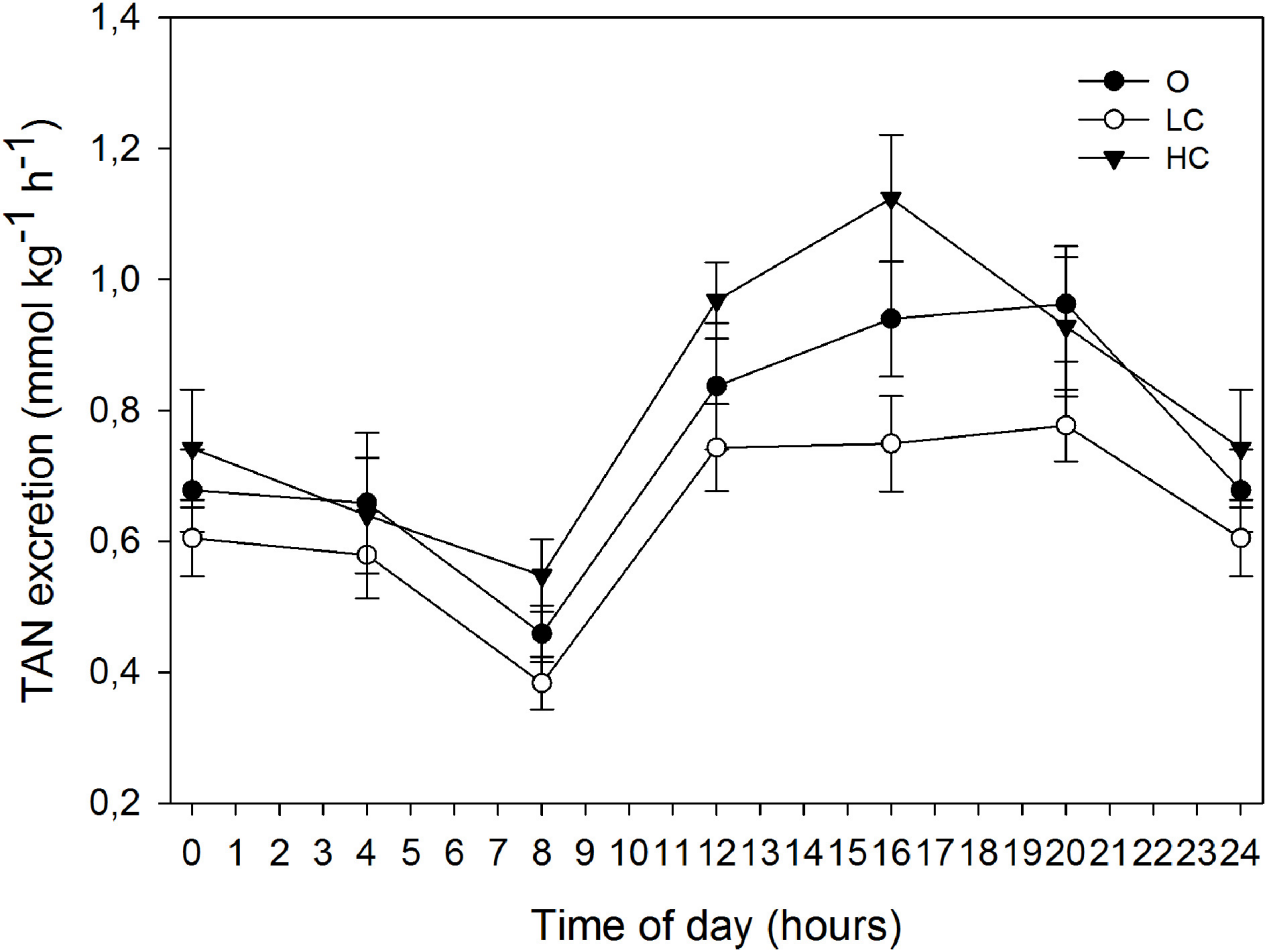
**Daily patterns of total ammonia nitrogen (TAN) excretion at different water current velocities**. Values are mean (±SE) for 3 days of measurements (days 78, 80, and 82 of the growth trial). High current, HC = 1 BL s^−1^; low current, LC = 0.5 BL s^−1^; and O = no current (*n* = 4).

The rate of TAN excretion over the six daily periods of measurement followed a similar circadian pattern as the oxygen consumption (Figure 4). All groups displayed a diurnal low at 8:00, immediately before the onset of feeding, after which TAN excretion rates began to increase. Each group showed a different progression in the three measuring periods after the onset of feeding: LC remained stable ~0.7 mmol kg^−1^ h^−1^, O steadily increased between 8:00 and 20:00, while HC peaked at 16:00 at ~1.1 mmol kg^−1^ h^−1^ and then decreased at 20:00 to a similar rate as O (~0.9 mmol kg^−1^ h^−1^). The *M*NH_3_-N (mmol kg^−1^ day^−1^, mean ± SE) for HC, LC and O was: 19.8 ± 1.05, 15.3 ± 0.50, and 18.2 ± 1.29, respectively. *M*NH_3_-N was significantly higher for HC than for LC.

The NQ assumes that the samples are representative of daily oxygen consumption and ammonia excretion. The mean (± SE) *M*O_2_ and *M*NH_3_ calculated from the average of the 3 days of sampling, as well as the calculated NQs, are given in Table 2. The oxygen consumption showed the same trend as throughout the remainder of the growth trial, with HC having a significantly higher rate than LC and O. The NQ for the O group was elevated, but not significantly so. The proportion of protein used for respiration was calculated from each NQ and are shown in Table 2.

**Table 2:** **Energetic parameters in fish reared under three different current speeds, high current, HC = 1 BL s^−1^; low current, LC = 0.5 BL s^−1^; and O = no current (mean ± SE, *n* = 4, except for O, where *n* = 3 in O_2_ consumed, E dissipated, E allocated and gross cost of growth)**.

	**O**	**LC**	**HC**
Mass gain (g fish^−1^)	99.60 ± 0.85	101.1 ± 0.43	100.5 ± 0.52
Time required (days)	28.3 ± 0.48	28.8 ± 0.25	30.3 ± 0.48
SGR (% day^−1^)	1.18 ± 0.01^a^	1.18 ± 0.02^a^	1.12 ± 0.02^b^
Total feed intake (g kg^−1^)	285.0 ± 5.49^a^	288.6 ± 3.03^a^	304.3 ± 4.35^b^
Total E intake (kJ kg^−1^)	6981.3 ± 134.44^a^	7070.5 ± 74.16^ab^	7455.6 ± 106.54^b^
Total O_2_ consumed (mmol kg^−1^)	3730.6 ± 273.11^a^	4153.5 ± 210.52^a^	5304.9 ± 182.70^b^
Total E dissipated for metabolism (kJ kg^−1^)	1622.8 ± 118.80^a^	1806.8 ± 91.58^a^	2307.6 ± 79.48^b^
%E dissipated for metabolism	23.6 ± 1.72^a^	25.6 ± 1.26^a^	30.9 ± 0.90^b^
%E allocated for growth	76.4 ± 1.72^a^	74.4 ± 1.26^a^	69.1 ± 0.90^b^
Total E allocated for growth (kJ kg^−1^)	5265.4 ± 166.70	5263.8 ± 105.29	5147.9 ± 91.81
Gross cost of growth (kJ g^−1^)	20.60 ± 0.16^a^	20.86 ± 0.30^a^	22.11 ± 0.28^b^

*The parameters were calculated per tank (kg biomass), except for SGR and gross cost of growth (see below). Superscripts indicate significant differences between treatments*.

## Discussion

### Growth performance

Studies on Arctic charr (*Salvelinus alpinus* Linnaeus, 1758) (Christiansen et al., 1989, 1992; Christiansen and Jobling, 1990), Atlantic salmon (*Salmo salar* Linnaeus, 1758) (Totland et al., 1987; Jørgensen and Jobling, 1993; Jørgensen et al., 1996) and brook charr (*Salvelinus fontinalis* Mitchill, 1814) (East and Magnan, 1987) have obtained positive effects on growth and feed conversion ratio, when fish are reared at moderate current speeds and fed to satiation. The coincidence of U_OPT_ with optimal swimming speed for growth appears to be the case for many species, including the salmonids (Davison and Herbert, 2013), although for rainbow trout, research on exercise induced promotion of growth has not yielded unequivocal results. So the question is whether rainbow trout is the odd one out or whether other things factor in. In the present study, the highest water velocity used was 1 BL s^−1^ which is considered to be the most energetically efficient swimming speed for rainbow trout with a mass of ~250 and reportedly the optimal for growth (Webb, 1971; Weihs, 1973; Walker and Emerson, 1978). The fish in this study were smaller than this size and were therefore in fact swimming well below their U_OPT_, emphasizing the need to consider what the U_OPT_ is for the size of fish being used.

Houlihan and Laurent (1987) did obtain higher growth rates in trout reared at 12°C swimming at 1 BL s^−1^ compared to non-swimming fish. These fish were fed to satiation, with no further report on the specific feed intake. McKenzie et al. (2012) found no positive effects on growth or feed conversion when feeding was limited to 1% at 0.9 BL s^−1^ and 14°C.

The present study found a significant negative effect of exercise at a current of 1 BL s^−1^ on both SGR and feed conversion ratio. The fish were reared in circular tanks, which dictated a swimming pattern with a turning component included at all times rather than a simple straight line motion. At the same time, a circular swimming canal will dictate a velocity gradient from outer to inner wall, and a drag effect along the tank walls. Although we did not systematically quantify these effects, any gradient or wall effect did not have any apparent effect, and fish positioned themselves evenly distributed in the water column and along the width of the canal. With the exception of Totland et al. (1987), all of the above mentioned studies employed circular rearing tanks, and the spatial properties of the rearing system seems an unlikely explanation for the differences in the results. Rather, the use of a restricted feeding regime may be an explanation for the poor SGR and FCRs obtained in this study, as was also observed in the study by McKenzie et al. (2012). When fish are fed *ad libitum*, it is likely that they are in energetic surplus (McKenzie et al., 2012), so that the increased cost of swimming does not limit growth. In that situation, the beneficial effects of water current, such as inhibition of aggressive behavior or erratic activity, can be of adequate magnitude to be reflected in growth.

### Routine metabolism

The rates and daily patterns of oxygen uptake were in line with previous investigations (McKenzie et al., 2012). There was a clear increase in routine activity when lights were turned on, that was reflected in the consumption of oxygen in the tanks. When feeding began, MO_2_ furthermore increased. This postprandial increase in metabolic rate corresponds to the specific dynamic action (SDA) and is believed to be associated with digestion, absorption, and protein assimilation (Jobling and Davies, 1980; Alsop and Wood, 1997; Owen et al., 1998; Seth et al., 2009). The HC group had a markedly higher RMR throughout the whole day and on average for the entire experiment.

### Bioenergetic budgets

The lower SGR of the HC group resulted in an increase in the number of days required to achieve an average mass gain of 100 g. Since all treatments had a similar intake of dietary energy, and assuming that digestibility of nutrients was equal between treatments, the decrease in SGR can be assigned to the increase in energy expenditure on routine activity. These findings demonstrate that rainbow trout forced to swim at 1 BL s^−1^, have a greater requirement for maintenance energy, which they must fuel by oxidizing a significantly higher fraction of their ingested energy at the cost of somatic growth. It is commonly accepted that exercising salmonids if fed to satiation, show faster growth than still water controls since they display an increase in appetite; however, when smaller rations are employed, growth rates are not improved (Davison, 1989). In addition to increase feed intake, this improved growth appears to be also mediated by improved nutrient retention, as shown by Grisdale-Helland et al. (2013) who also observed higher maintenance energy requirements in Atlantic salmon swimming at speeds of 1.1 vs. 0.3 BL s^−1^, that did not manifest in different growth rates despite being maintained on rations of 0.9% BM d^−1^.

### Ammonia excretion and protein use

Daily fluctuations in TAN excretion are linked to feeding and the maximum postprandial excretion, and are typically 30–67% higher than the daily mean (Thorarensen and Farrell, 2011), which is in line with the observations in the present study. The average daily ammonia excretion was significantly higher in HC than in LC. This shows that absolute rates of protein turnover increase with increasing current speeds. However, the fact that *M*NH_3_-N in O was not significantly different from the groups with water current suggests that we should be cautious in the conclusions we draw.

The NQ is a measure of protein utilization obtained from the relationship between oxygen consumption and nitrogenous waste excretion. The results of the present study show average rates of protein use to fuel metabolism is slightly but not significantly higher in the O group (~60%) while the LC and HC groups used less than 50%. These tendencies in the NQ values are brought on by changes in *M*O_2_ and less by *M*N. NQ values for fish are highly variable, ranging between 14% and 90% in different species (Van Waarde, 1983; Weber and Haman, 1996; Lauff and Wood, 1996b; McKenzie et al., 2007). In fasted salmonids protein has been estimated to contribute 14–36% but these values may increase up to close to 100% in actively feeding fish (Kajimura et al., 2004). The values found in this study are higher than those obtained by Lauff and Wood (1996a,b) in studies of instantaneous fuel usage during aerobic swimming in fasted juvenile rainbow trout (22–45% in untrained fish and 17–36% in fish trained at 2.1 BL s^−1^), but are in agreement with the results obtained by Alsop and Wood (1997) in trout fed 1% body mass per day (~40%) and fed to satiation (~70%). The abovementioned studies employed an instantaneous approach based on respirometry and measurement of excreted ammonia-N and urea-N. However, the use of summed ammonia-N and urea-N is thought to underestimate the contribution of protein toward metabolism (Kajimura et al., 2004) because it does not consider other oxidized N-products resulting from protein catabolism. On the other hand, NQ based on total-N excretion may overestimate protein oxidation because total-N includes a significant proportion of non-oxidized N-products such as proteins excreted in mucus.

A complete analysis of fuel usage would require the measure of carbon dioxide excretion rates and calculation of respiratory quotients besides nitrogen quotients, and should be conducted on fed as well as fasted fish. A more detailed understanding of instantaneous fuel use is key to explaining exercise induced, non-feed intake related, effects on growth. While the present study does not allow a full discussion on the partition of metabolic fuels between protein, lipid and carbohydrates, the results indicate a trend for a decrease in the relative contribution of protein when a current is present. This suggests that there may be a decrease in protein catabolism during aerobic swimming, which is in accordance with the findings of Alsop and Wood (1997) and Lauff and Wood (1996a,b), however, it is evident that for trout and salmon this depends on several factors, in particular feed intake and dietary macronutrient content. This is exemplified by the work of Felip et al. (2012) who observed significant increases in nitrogen recovery in white and red muscle protein when rainbow trout were forced to swim while fed a diet with 30% digestible carbohydrate. This effect was brought on partly by the aforementioned increase in glucose oxidation, but also by an increased lipogenesis which was subsequently used to fuel swimming. When salmon are reared on commercial diets with lower levels of carbohydrate (e.g., 12–17%) this effect has not been observed.

## Conclusion

The present study has contributed to illustrate the complexity of the physiological responses of rainbow trout to a moderate exercise. It seems that swimming against a current does not confer an energetic advantage *per se*; rather it may even negatively affect growth performance. The proposed explanation for these results is an exacerbation of the metabolic expenditure relative to the total energy available in exercised fish, possibly due to a restricted feeding regime. While imposing an exercise regime on fish carried a significant increase in metabolic cost, calculated nitrogen revealed a tendency toward a reduced relative use of protein to fuel metabolism in exercised fish. The use of lipid and carbohydrate to fuel exercise metabolism during fasting and feeding should be quantified as a logical next step. The results of this study also lend support to previous findings of a reduction in minimum metabolic requirements induced by exercise. This effect deserves further investigation.

### Conflict of interest statement

The authors declare that the research was conducted in the absence of any commercial or financial relationships that could be construed as a potential conflict of interest.
